# Ceramides and Sphingosino-1-Phosphate in Obesity

**DOI:** 10.3389/fendo.2021.635995

**Published:** 2021-05-13

**Authors:** Ilona Juchnicka, Mariusz Kuźmicki, Jacek Szamatowicz

**Affiliations:** Department of Gynecology and Gynecological Oncology, Medical University of Białystok, Białystok, Poland

**Keywords:** ceramides, sphingolipids, S1P, obesity, adipose tissue

## Abstract

Obesity is a growing worldwide problem, especially in developed countries. This disease adversely affects the quality of life and notably contributes to the development of type 2 diabetes, metabolic syndrome, and cardiovascular disorders. It is characterised by excessive lipids accumulation in the subcutaneous and visceral adipose tissue. Considering the secretory function of adipose tissue, this leads to impaired adipokines and cytokines release. Changes in adipose tissue metabolism result in chronic inflammation, pancreatic islets dysfunction and peripheral insulin resistance. In addition to saturating various adipocytes, excess lipids are deposited into non-adipose peripheral tissues, which disturbs cell metabolism and causes a harmful effect known as lipotoxicity. Fatty acids are metabolised into bioactive lipids such as ceramides, from which sphingolipids are formed. Ceramides and sphingosine-1-phosphate (S1P) are involved in intracellular signalling, cell proliferation, migration, and apoptosis. Studies demonstrate that bioactive lipids have a crucial role in regulating insulin signalling pathways, glucose homeostasis and β cell death. Data suggests that ceramides may have an opposite cellular effect than S1P; however, the role of S1P remains controversial. This review summarises the available data on ceramide and sphingolipid metabolism and their role in obesity.

## Introduction

Obesity is an increasingly common phenomenon. The global prevalence of obesity has almost tripled in the last 40 years (1). Obesity is a risk factor for several serious chronic diseases. It contributes to developing certain types of cancer, cardiovascular complications, insulin resistance, metabolic syndrome, type 2 diabetes, asthma, hepatic and renal dysfunction, infertility and sleep disturbances (2).

There are no doubts about the harmfulness of obesity, but, at the same time, the power of adipose tissue is becoming clear. Adipose tissue is the largest endocrine organ built from diverse types of cells. The primary cells are adipocytes. In addition to adipocytes, there are pre-adipocytes, mesenchymal cells, fibroblasts, endothelial cells and immune cells (3). There are two basic types of adipose tissue. The dominant is white adipose tissue (WAT). Due to the richness of the cells from which it is produced, WAT performs many different functions. First, it serves as a vast energy store that regulates fatty acid homeostasis. During excessive food intake, free fatty acids (FFA) accumulate in WAT as triacylglycerols (TAG). Another function of WAT is the secretion of adipokines, including adiponectin, leptin, resistin, apelin visfatin and cytokines like tumour necrosis factor α (TNFα), interleukin-6 (IL-6) and plasminogen activator inhibitor-1 (PAI-1) (4, 5). There are two types of WAT. The subcutaneous adipose tissue (SAT) is located under the dermal layer. Visceral adipose tissue (VAT) is located around the internal organs. The two classes have similar morphological structures, but the most important aspect is their metabolic diversity (5).

In obesity, the phenomena of hypertrophy (increased adipocyte size) and hyperplasia (increased adipocyte number) are present. Hypertrophy is harmful and associated with the reduced release of adiponectin, increased release of pro-inflammatory cytokine and fatty acid, impaired insulin sensitivity, hypoxia, and immune cell activation. In contrast, hyperplasia has the opposite effect (6). Adipocytes are overloaded and lose their lipid storage capacity. They can store an excessive amount of fat and energy. Still, in the case of unnecessarily high food intake, a release of FFA from the adipocytes occurs which then is being stored in non-adipose tissue. This release has an adverse effect on the human body, called lipotoxicity (7).

Brown adipose tissue (BAT) is located supraclavicularly and paravertebrally. BAT controls the body’s temperature by activating an uncoupling protein 1 (UCP1) located in the mitochondrial membrane. The UCP1 is stimulated by exposure to cold and immediately uses energy and conducts heat (6, 8). A meal rich in carbohydrates and essential macronutrients is also a stimulus to UCP1 for thermogenesis (9).

Additionally, a tissue that is a combination of both is described as a beige, so-called browning adipose tissue. It could emerge *de novo* from the progenitor cells or SAT under the influence of stimuli such as cold or by the activation of the β3-adrenergic receptor, for example, by catecholamines. Beige adipocytes contain UCP1 in ten-fold lower concentration than brown tissue. Thanks to brown and beige adipocytes’ unique ability to generate energy and thus consume glucose and triglycerides, their protective effect against obesity is recognised (8, 10). The beneficial influence of browning WAT and energy expenditure may be considered one of the therapeutic goals in obesity treatment.

This paper aims to review the current state of knowledge on ceramides and S1P and their link to obesity. Considering the rapid development in this field of science, a summary of the available data could prove valuable.

## Ceramides

Ceramides generally contain the sphingoid 18 carbon chain base with a 14 to 30 carbon length fatty acyl chain. They can be modified to produce more complex sphingolipids like sphingomyelin, galactosylceramide, glucosylceramide, ganglioside and globoside (11). As the primary components of the plasmatic membrane, ceramides have an impact on cell membrane properties. The potential for their redistribution within the membrane leads to a change in its activity and response to enzymes (12). When substrates for synthesis are provided in excess, ceramides may accumulate in tissues. There are three biosynthesis pathways leading to sphingolipid formation that have ceramides as their metabolic hub.

## Sphingolipids Synthesis

**Figure 1:**
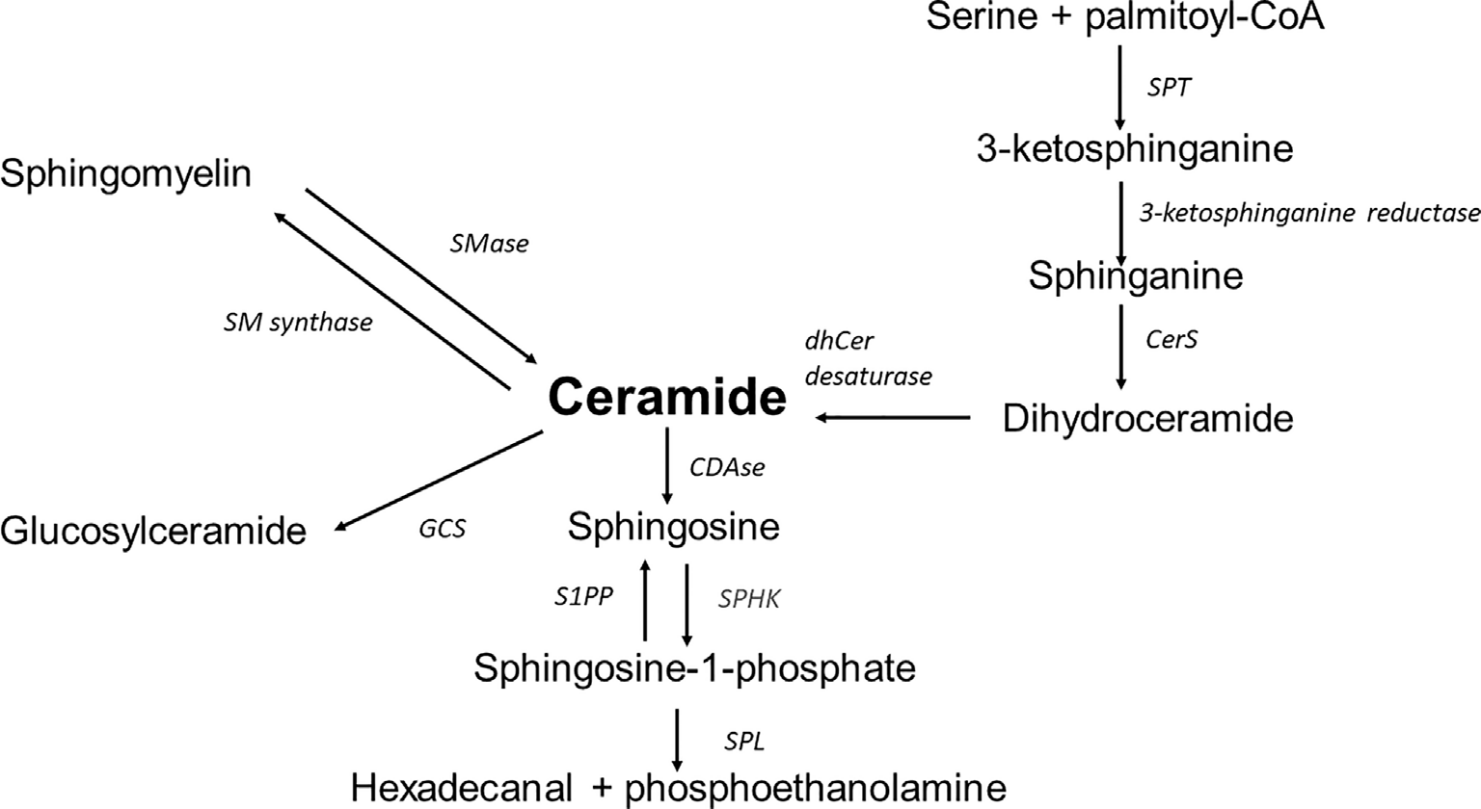
Overview of sphingolipid metabolism; SPT, serine palmitoyltransferase; CerS, ceramide synthase; dhCer desaturase, dihydroceramide desaturase; S1PP, sphingosine-1-phosphate phosphatase; SPHK, sphingosine kinase; SPL, sphingosine-1-phosphate lyase; CDase, ceramidase; SMase, sphingomyelinase; SM synthase, sphingomyelinase synthase; GCS, glucosyl-ceramide synthase.

Sphingolipids are a diverse lipid class built of an amino alcohol, sphingosine or dihydrosfingosine (sphinganine) as an N-acylated backbone. Due to the modification of this basic structure, the identification of a family of numerous sphingolipids such as ceramides, sphingomyelins, glycolipids, and gangliosides is possible. Structural variety is followed by a variety of multiple biological functions (11, 13). The synthesis of sphingolipids depends on many metabolic compounds exogenously delivered or transferred from sphingolipid turnover. There are three biosynthesis pathways (**Figure 1**).

1. The *de novo* pathway is placed in the endoplasmic reticulum (ER) and begins with the condensation of palmitoyl coenzyme A (CoA) and L-serine. Although palmitoyl-CoA and serine are preferred in this reaction, stearate or myristate and alanine or glycine can also be used. An enzyme mutation could cause the substrate shift (13). For example, alanine is used as a substrate in the serine palmitoyltransferase (SPT) mutation. As a result of the reaction, neurotoxic deoxysphingolipids are formed (14). Under normal conditions, the reaction generates 3-ketosphinganine by SPT. Subsequently, 3-ketosphinganine reductase is responsible for reducing 3-ketosphinganine to sphinganine, which is acetylated to dihydroceramide by ceramide synthase (CerS1-6). Dihydroceramide is oxidised by desaturase, which results in the formation of the ceramide. Ceramide synthase occurs in six isoforms in mammals, each of which creates a ceramide with a particular acyl chain length (C14:0-C30:0) (15). The specific location of those enzymes remained unclear. However, data indicates the ER as the primary site of CerS occurrence. Other localisations of CerS are mitochondria and the nucleus (16).
2. The *salvage pathway* is part of the second biosynthesis route. The ceramide is deacylated by ceramidases to produce sphingosine, which is phosphorylated by sphingosine kinases (SphK) to sphingosine-1-phosphate (S1P). As a result of further changes catalysed by S1P lyase, S1P is transformed into fatty aldehydes and ethanolamine phosphate, which become substrates for the cascade of enzymatic reactions from which fatty acyl-CoA is obtained. Another possible transformation of S1P is dephosphorylation by S1P phosphatase leading back to sphingosine and then by ceramide synthase to ceramide (17).
3. The *Sphingomyelin pathway* takes place in the Golgi apparatus. Through the action of sphingomyelin synthase out of a ceramide the sphingomyelin (SM) is formed. Afterwards SM is transported to the plasma membrane. In the plasma membrane, the reaction is reversible, with SM transforming back to ceramide using sphingomyelinase. Then, ceramides can be deacylated by ceramidase to sphingosine, which can be phosphorylated by SphK to S1P. SM can be transported to the lysosome from the plasma membrane, where the cascade of reactions is the same; it progresses from SM through ceramide to sphingosine (18).

## Ceramides in Skeletal Muscles

In skeletal muscles, which play an important role in glucose uptake, the accumulation of the ceramides is strongly related with insulin resistance and diabetes (19). Elevated total ceramides content disturbs the insulin pathway mainly at the level of kinase B (Akt) through the activation of protein phosphatase 2A (PPA2) which keeps Akt unphosphorylated, thereby inhibiting further steps of the pathway. Consequently, the glucose transporter type 4 (GLUT4) translocation to the plasma membrane is impaired and the muscles are not efficient in glucose uptake (20).

Data demonstrated that decreasing ceramide concentration in skeletal muscles eliminates the deleterious effect and improves glucose tolerance. Inhibition of SPT by myriocin treatment prevents ceramide-induced glucose intolerance and insulin resistance by enabling Akt phosphorylation (21). Moreover, research focused on the ceramide transport from the ER to the Golgi apparatus through the ceramide transporter (CERT) showed that CERT overexpression decreased ceramide accumulation in muscles thus improving insulin signalling (22).

## Ceramides in Adipose Tissue

The content of ceramides in obesity is different in every type of investigated adipose tissue. VAT shows a strong positive correlation with metabolic diseases and cardiovascular complications (23). Studies have shown that VAT shows a greater ability to change tissue metabolism than SAT. The proximity of internal organs enables VAT to modulate their metabolism easily. Data showed elevated levels of C14-Cer, C16-Cer and C18:1-Cer in the obese non-diabetic group compared to the lean non-diabetic group. Interestingly, further growth of C18-Cer and C24:1Cer was proven in the third obese diabetic group. This may indicate the direct participation of those components in the development of diabetes (24). The accumulation of ceramides in VAT was also observed in the metabolic syndrome (25). In both experiments, total ceramide concentration was increased in the VAT of obese subjects compared to lean non-diabetic patients. The enhanced ability to gather ceramides in VAT adipocytes may cause a weak insulin response, reduced lipogenesis and fewer lipid droplets than SAT. Visceral obesity positively correlates with glucose level, insulin resistance, TAG and cholesterol concentration (26).

However, the total ceramide content was also measured in the SAT. It proved to be elevated in lean patients compared to obese patients (27) and obese with metabolic syndrome (25). Another study reports a decreased total ceramide level in lean, healthy patients compared to obese patients (24). The ambiguity of the results is due to the difference in the location of the collected tissues. The SAT was taken from the abdomen (25, 27) and the sternum (24). Researchers suggest that excessive food intake leads to hyperplasia in lower body SAT and hypertrophy in upper body SAT (26). Such a high variability within one tissue prompts further research to deepen the understanding of the pathomechanisms occurring in it and the factors that may modulate its glucose and lipid metabolism.

It is considered that accumulation of ceramides in the adipose tissue negatively affects the inhibitory effect of insulin on hormone-sensitive lipase (HSL) activity. Under physiological conditions HSL stimulates lipolysis but postprandial insulin release has a known HSL inhibitory effect. It has been demonstrated that in obesity accompanied by insulin resistance the accumulated ceramides influence insulin causing a decreased HSL inhibition. Consequently, resulting in an increased FFA concentration in the plasma (28, 29).

In the WAT isolated from obese humans and rodents, the CerS6 was significantly increased, and the correlation between BMI, hyperglycaemia and body fat content was favourable compared to lean subjects. An experiment with knockdown CerS6 mice was performed, demonstrating a reduced concentration of C16:0 in WAT, BAT and liver compared to control mice. The concentration in skeletal muscles was at the same level. However, most relevant was that in mice with a deletion of CerS6 despite being HFD-fed, a reduced body mass and body fat content, improved glucose metabolism, reduced adipocyte size and decreased leptin concentration were observed. Research revealed that deletion of CerS6 in BAT appears to be crucial in improving glucose homeostasis by increasing mitochondrial β-oxidation (30, 31). These data are strong evidence that C16:0, a product of CerS6, is a significant factor in the development of obesity and its related complications.

Data demonstrated that sensitising tissues to insulin is possible by inhibiting *de novo* ceramides synthesis using myriocin, which blocks SPT activity. A study was conducted on mice in which insulin resistance was induced by HFD. After *in vivo* myriocin treatment, a decreased Cer and diacylglycerol (DAG) concentrations in VAT and SAT were measured. Furthermore, a strong correlation between total ceramide content in AT and adiponectin secretion (negative) and TNFα levels (positive) was observed (28).

## Lipotoxicity in Obesity

Lipotoxicity is caused by excessive nutrient intake and increased lipid levels in the bloodstream. This process leads to defective lipid oxidation, increased ceramide formation and accumulation of bioactive lipids in organs and tissues. Lipotoxicity has a substantial impact on pancreatic β-cells by impairing glucose-stimulated insulin secretion (32). Most significant is that ceramides contribute to β-cell apoptosis by releasing cytochrome c from the mitochondria and activating the apoptotic cascade in the lipotoxicity process (33). It has been shown that palmitate harms the insulin promoter and blocks insulin gene expression in rat pancreatic islets. The entire process is accompanied by *de novo* production of ceramides (34). However, fumonisin B1, a ceramide synthetase inhibitor, may be able to stop the harmful effects of palmitic acid and ceramides (32). Inhibition of ceramide synthesis prevents the harmful effects of palmitate on insulin gene expression (34).

Recently the promotion of lipotoxicity was indicated by activation of SphK2 in β-cells. The excess of palmitic acid present in obesity predisposes to redistribution of SphK2 from the nucleus to the cytoplasm; this signal to relocate is responsible for β-cell lipotoxicity. The lack of SphK2 ameliorates insulin secretion by protecting β-cells against apoptosis (35).

Lipotoxicity impairs the proper functioning of the liver, kidneys, and muscles, including cardiomyocytes. High levels of saturated fatty acids result in mitochondrial membrane superoxide and reactive oxygen species (ROS) production, causing oxidative stress with a reduced antioxidant response (36). Lipotoxicity leads to ER stress, which plays an essential role in insulin resistance and cell death. Interestingly, increased ER stress in the hypothalamus modulates the sympathetic response of BAT, leading to reduced thermogenesis and weight gain (37). Lipotoxicity is a destructive process that can contribute to the development of metabolic disorders.

## Sphingolipids and Adiponectin

Adiponectin controls lipid metabolism and glucose homeostasis by increasing glucose consumption in skeletal muscles. Adiponectin works with two receptors, AdipoR1 and AdipoR2. AdipoR1/2 have ceramidase activity by binding and hydrolysing the ceramide to FFA and sphingosine, substrates in S1P production (38). As a result, ceramide levels are reduced, and glucose utilisation and tissue insulin sensitivity are improved. Data confirms that this binding leads to lipid oxidation, mitochondrial biogenesis, and anti-apoptotic modifications. Lack of those receptors may be the reason for metabolic dysfunction (38, 39). It proves that adiponectin receptors may be crucial in bioactive lipid balance.

A study conducted on mice showed that increased concentrations of circulating adiponectin negatively correlates with ceramide levels. Moreover, it enhances insulin sensitivity caused by the fibroblast growth factor (FGF21), which stimulates adiponectin secretion. FGF21 treatment in mice showed an increased adiponectin secretion, reducing the accumulation of ceramides in tissues prevents lipotoxicity. In obese and diabetic mice, the FGF21 reduced blood glucose concentration and improved insulin sensitivity. However, adiponectin knockout mice showed no positive changes after FGF21 stimulation (40). Noteworthy is that the ablation of SPT also increased the release of FGF21 and improved metabolism (41).

## Sphingosine-1-Phosphate

**Figure 2:**
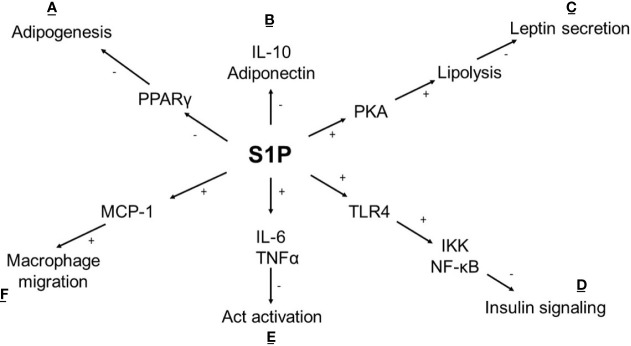
Metabolic effect of S1P in adipocyte. **(A)** S1P inhibits adipogenesis by affecting the expression of transcriptional factor PPARγ (45); **(B)** S1P reduces the anti-inflammatory response (IL-10) and adiponectin synthesis which is accompanied by **(E)** an increased proinflammatory cytokine levels (IL-6, TNFα) lead to the activation of PP2A leaving Akt dephosphorylated and inactive (46); **(C)** In cAMP/PKA dependent pathway S1P stimulates lipolysis and inhibits insulin-mediated leptin synthesis (47); **(D)** S1P intensifies TLR4 activation cause impaired insulin signalling *via* IKK- NF-κB axis (48); **(F)** S1P stimulates secretion of chemokine MCP-1 leads to increased macrophage migration (49); (+) activation, (-) inhibition, TNFα -tumor necrosis factor, IL-6, interleukin 6; IL-10, interleukin 10; Act, protein kinase 3; TLR4, Toll like receptor 4; IKK, inhibitor kappa kinase; NF-κB, nuclear factor κB; PKA, protein kinase A; PPARγ, peroxisome proliferator-activated receptor gamma; MCP-1, monocyte chemoattractant protein-1.

Ceramides are the primary source of *de novo* S1P synthesis through a process of sphingosine diacylation. Diacylation is catalysed by two isoenzymes, SphK1 (located in the cytoplasm) and SphK2 (located in the nucleus, mitochondria, and ER), both widely expressed in human tissues. S1P is a bioactive lipid that takes part in numerous cellular processes such as angiogenesis, cell growth, apoptosis and inflammation by binding to S1P_1-5_ receptors (42, 43). S1P has anti-apoptotic properties, enhances insulin sensitivity and reduces immune response (44) (**Figure 2**). The study conducted on HFD mice demonstrated a positive influence of the S1P analogue on insulin signalling and reduced leukocyte accumulation in adipose tissue (50). On the other hand, an increased level of S1P is observed in the SAT of obese diabetic patients, and a negative effect on insulin signalling is confirmed (24, 51, 52). The difference may depend on a non-specific affinity of S1P to the S1P receptor. It was surprising that in-vitro S1P interacts with CerS2 by a motif located in CerS2, which is similar to the S1P receptor causing the inhibition of CerS2. This could explain the antagonistic effect of S1P on ceramides (53).

Another critical point is the tissue of action, the type of SphK isoenzymes and their expression. In muscles, S1P leads to Akt activation, responsible for improved insulin response by increasing glucose uptake and glycogen synthesis (54). By contrast, in adipose tissue, S1P inhibits Akt activation after insulin stimulation (49). In both tissues, the increased expression of SphK1 was observed. No data indicate the participation of SphK2 in the impaired insulin response of the described tissues. The SphK1 and SphK2 effect on pancreatic β cell activity is believed to be antagonistic. Saturated fatty acids stimulate the SphK1/S1P axis by inhibiting lipotoxicity-induced β cell apoptosis (55).

In contrast, the SphK2 under the lipotoxic condition passes to the cytoplasm, promoting the apoptosis of β-cells and leading to impaired glucose homeostasis (35, 56). Surprisingly, the positive role of SphK1 and SphK2 was observed after exposure to high glucose levels. This resulted in increased S1P production and elevated insulin synthesis and secretion, leading to reduced serum glucose level (57).

Another controversy is the influence of S1P on inflammatory processes. In obesity a chronic inflammation state is present. As an endocrine organ, adipose tissue secretes adipokines and chemokines such as pro-inflammatory cytokines. HFD results in an accumulation of DAG and ceramides in the adipose tissue and, simultaneously, leads to an increased SphK1 expression and conversion of ceramide to S1P. S1P promotes pro-inflammatory cytokine expression (TNFα, Il-6) and secretion in adipose tissue Studies have shown that the SphK1 deficiency in DIO mice resulted in enhanced adipogenesis and anti-inflammatory cytokine expression (Il-10). Further, glucose tolerance and insulin sensitivity in muscle and adipose tissue were improved (49).

In contrast, endogenous S1P has a protective impact on β-cells against cytokine-induced apoptosis in rat islets (58). The difference in the S1P action is determined by the protein with which S1P is combined. In the bloodstream, S1P is transported by albumin (~35%) or apolipoprotein M (apoM) combined with HDL cholesterol (~65%) (42). Albumin is a protein which binds many hydrophobic compounds in the bloodstream whereas apoM/HDL remains specific and probably is critical in biological response. The S1P/apoM/HDL complex reveals an anti-inflammatory effect on endothelial cells and helps to maintain vascular integrity, which is the aim of vascular disease treatment (59).

## Conclusions

Obesity is increasingly a global problem. The basis of complications related to obesity is adipose tissue overgrowth and accumulation of bioactive lipids. Their role seems to be crucial in insulin resistance, diabetes, hypertension and dyslipidaemia development (60).

Treatment that reduces sphingolipid levels in the bloodstream is a promising method in fighting obesity and other related diseases (61, 62). Nevertheless, continuous identification of the mechanisms controlled by bioactive lipids is essential. The cognition of modulation of immune response, thermogenesis, glucose, and lipid homeostasis by sphingolipids will be crucial in the upcoming years.

## Author Contributions

IJ wrote the draft of the manuscript. MK reviewed and edited. JS reviewed and edited. All authors contributed to the article and approved the submitted version.

## Funding

The study was supported by funds from Medical University of Białystok, Poland SUB/1/DN/19/003/1129 and SUB/1/DN/19/001/1129.

## Conflict of Interest

The authors declare that this research was conducted in the absence of any commercial or financial relationships that could be construed as a potential conflict of interest.
